# Genetic Diversity within a Collection of Italian Maize Inbred Lines: A Resource for Maize Genomics and Breeding

**DOI:** 10.3390/plants13030336

**Published:** 2024-01-23

**Authors:** Anna Maria Mastrangelo, Hans Hartings, Chiara Lanzanova, Carlotta Balconi, Sabrina Locatelli, Helga Cassol, Paolo Valoti, Giuseppe Petruzzino, Nicola Pecchioni

**Affiliations:** 1 CREA-Centro di Ricerca Cerealicoltura e Colture Industriali/Research Centre for Cereal and Industrial Crops, SS 673 Metri 25200, 71122 Foggia, Italy; pinospazio@gmail.com (G.P.); nicola.pecchioni@crea.gov.it (N.P.); 2 CREA-Centro di Ricerca Cerealicoltura e Colture Industriali/Research Centre for Cereal and Industrial Crops, Via Stezzano 24, 24126 Bergamo, Italy; hans.hartings@crea.gov.it (H.H.); chiara.lanzanova@crea.gov.it (C.L.); carlotta.balconi@crea.gov.it (C.B.); sabrina.locatelli@crea.gov.it (S.L.); helga.cassol@crea.gov.it (H.C.); paolo@valoti.it (P.V.)

**Keywords:** maize, germplasm collections, genetic diversity

## Abstract

Genetic diversity is fundamental for studying the complex architecture of the traits of agronomic importance, controlled by major and minor loci. Moreover, well-characterized germplasm collections are essential tools for dissecting and analyzing genetic and phenotypic diversity in crops. A panel of 360 entries, a subset of a larger collection maintained within the GenBank at CREA Bergamo, which includes the inbreds derived from traditional Italian maize open-pollinated (OP) varieties and advanced breeding ones (Elite Inbreds), was analyzed to identify SNP markers using the tGBS^®^ genotyping-by-sequencing technology. A total of 797,368 SNPs were found during the initial analysis. Imputation and filtering processes were carried out based on the percentage of missing data, redundant markers, and rarest allele frequencies, resulting in a final dataset of 15,872 SNP markers for which a physical map position was identified. Using this dataset, the inbred panel was characterized for linkage disequilibrium (LD), genetic diversity, population structure, and genetic relationships. LD decay at a genome-wide level indicates that the collection is a suitable resource for association mapping. Population structure analyses, which were carried out with different clustering methods, showed stable grouping statistics for four groups, broadly corresponding to ‘Insubria’, ‘Microsperma’, and ‘Scagliolino’ genotypes, with a fourth group composed prevalently of elite accessions derived from Italian and US breeding programs. Based on these results, the CREA Italian maize collection, genetically characterized in this study, can be considered an important tool for the mapping and characterization of useful traits and associated loci/alleles, to be used in maize breeding programs.

## 1. Introduction

Maize (*Zea mays* subsp. *mays*) is one of the most important agricultural crops worldwide. Northern Italy is one of the core areas for maize cultivation and production in Europe, with a grain yield and corncob mix of more than 4.7 million tons per year [1]. In historical times, maize cultivation was boosted through the diffusion of maize germplasm, which was better adapted to European conditions, especially to longer photoperiods, after the first introduction of genetic materials from the Caribbean [2]. Since then, farmers have developed numerous landraces by crossing different ecotypes and have adapted to specific environmental conditions and traditional farming systems [3]. At the end of the eighteenth century, maize reached a very similar level of production to that of wheat in many regions of northern Italy, where most of the maize genotypes were grown from open-pollinated (OP) seeds, until the first part of the last century. Then, after the discoveries on inbreeding and heterosis in the early 1900s, open-pollinated varieties were gradually replaced by double- and single-cross hybrids, which played a major role in increasing grain yield since the late 1930s [1,4]. In order to avoid the extinction of landraces and the loss of precious germplasm, a survey of maize varieties, diffused in Italy, was carried out in 1949–1950 by the Regional Inspectorates of Agriculture (*Ispettorati Provinciali dell’Agricoltura*). In 1954, researchers in the Experimental Station for Maize Cropping (*Stazione Sperimentale per la Maiscoltura*) of Bergamo (director: L. Fenaroli) started a systematic acquisition of Italian maize germplasm, through a national sampling program of “Indentata” and “Indurata” types, under the aegis of the Italian Ministry of Agriculture. With the cooperation of the *Ispettorati Provinciali dell’Agricoltura*, samples of different populations were taken and moved to Bergamo for reproduction and classification studies [3,5]. *Zea mays* var. *indentata* (dent) and *Z. mays* var. *indurata* (flint) are types of maize commonly cultivated in Europe. The main differences between them are at the level of the morphology and anatomy of grain. Flint types are characterized by a vitreous endosperm texture, round and large kernels, and a thick layer of hard endosperm on the crown, while dent types have mostly soft endosperm and a thin layer of hard endosperm only on the dorsal side of the grain, causing typical depression in the distal face [6]. Studying the extent of the genetic diversity of germplasm collections is of paramount importance to understand their potential deployment in breeding programs. Moreover, it is the basis for genetic association studies with the aim of understanding the complex architecture of the quantitative traits of agronomic significance. However, until now, the Italian maize GenBank has only been partially characterized genotypically [7], leaving its genetic diversity largely unexplored.

Genotyping technologies have greatly improved in recent years due to the development of next-generation sequencing (NGS) procedures, allowing for a high-throughput and relatively cheap and rapid analysis of large maize collections. Two common approaches have been mostly used so far: single-nucleotide polymorphism (SNP) array platforms and the genotyping-by-sequencing (GBS) process, with other NGS methods growing on the market. In maize, an Illumina Infinium HD 50,000 SNP array, named MaizeSNP50, was developed by Ganal et al. [8] and has been extensively used for diversity and association studies [9,10,11]. Nevertheless, the maize genome size (2.4 Gb), the high level of diversity, and the low LD extent have favored the spread of platforms with a higher marker density. An Affymetrix Axiom 600,000 SNP array was therefore developed and used in association genetics [10,12,13] for the detection of selective sweeps [14]. SNP arrays offer great advantages in genetic analysis as they are fast and provide results on markers that can be easily compared across different germplasm collection studies. On the other hand, they present the drawback of being ‘closed’ tools, as they do not allow for the discovery of de novo SNPs. Moreover, they are characterized by some ascertainment bias in diversity analyses since the SNPs selected for developing arrays are derived from a fixed set of individuals that are different, for example, from those in the present panel. Therefore, some SNPs on the array can be noninformative on the panel under study or can show different allelic frequency profiles, compromising the ability of SNP arrays to provide an exact evaluation of the genetic diversity [15,16]. This limit is overcome by GBS, in which SNPs are determined in the exact genotype panel under study and are usually available at a lower cost than SNP arrays [17]. However, a good balance between the cost of the assay and the sequencing depth needs to be found, as the more extensive the coverage, and the higher the sequencing depth, the higher the quality of the markers discovered, and thus the higher the cost of the assay. Moreover, the sequencing depth needed can vary across the genome and even between individuals, so large portions of the genome could remain without successful SNP calls if the right read depth is not considered [18,19]. Fortunately, a part of the missing data present after GBS and filtering can be recovered through imputation with ad hoc methods [19,20,21].

A genotyping-based analysis of the genetic diversity of a panel of 360 lines, a subset of a larger collection preserved at the CREA Bergamo GenBank, which includes the accessions derived from traditional Italian maize varieties and advanced breeding lines, is presented in this study. The SNP dataset obtained following GBS was subjected to analyses including the measures of genetic diversity, linkage disequilibrium, and genotypic clustering with Bayesian methods and methods based on supervised machine learning approaches. The results herein reported show the genetic structure of this panel of traditional Italian lines, which can represent an important genetic tool for the future identification and study of loci and alleles associated with agronomically relevant traits through genome-wide association mapping studies (GWAS) and for their use in maize breeding.

## 2. Results

### 2.1. Genotyping of the Maize Collection

The genotypic characterization of the maize collection was carried out through the tunable genotyping-by-sequencing (tGBS^®^) method, a technology that, compared to conventional GBS, produces better results in terms of the number of on-target reads and the amount of missing data thanks to the additional steps leading to a reduction in genomic DNA [22]. The experiment was conducted with the restriction enzyme Bsp1286I (Freedom Markers, Data2Bio, Ames, IA, USA). Samples were sequenced using an Illumina HiSeq X instrument (Illumina, San Diego, CA, USA), and reads were aligned to the *Zea mays* AGPv4 (GCA_000005005.6) reference. A total of 2 × 671,777,289 reads were obtained, with 2 × 1,749,420 average reads per sample and the number of reads per sample ranging from 2 × 541 to 2 × 5,742,482. A total of 53.1% of reads showed a single unique alignment to the maize genome, allowing us to establish an initial dataset of 797,368 SNPs. The details about the number of quality-trimmed sequence reads and aligned reads obtained for each sample are provided in Table S1. 

After several quality-filtering steps (20% missing data, 20% heterozygous calls, and minimum allele frequency > 0.05), a total of 15,872 SNP markers were retained, with a mean of 1587.2 SNP markers per chromosome (from a minimum of 1189 SNPs on chromosome 6 to a maximum of 2315 SNPs on chromosome 1) and an average density of 7.56 SNPs per Mbp (Table 1). 

**Figure 1:**
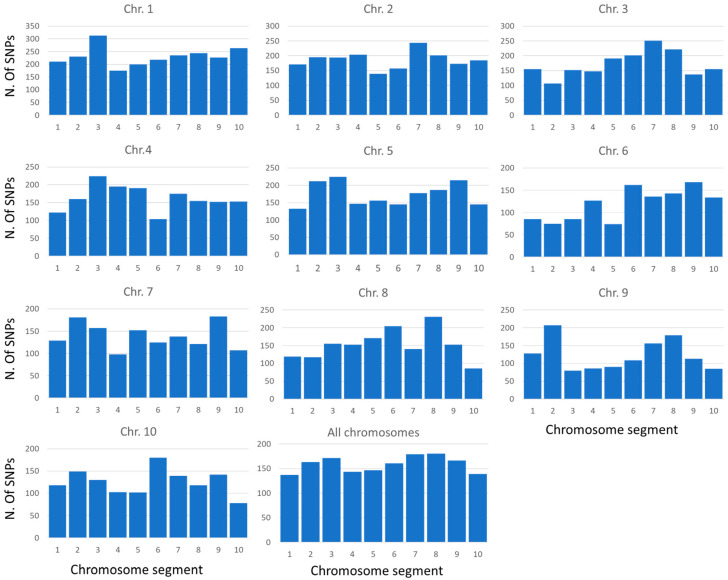
Number of SNPs per chromosome segment (each segment corresponding to the total length of the chromosome divided by ten), from proximal (1) to distal (10) regions, both for single chromosomes and as an average across all chromosomes.

SNPs showed a rather uniform distribution along chromosomes. In general, marker density results were lower in pericentromeric regions than in proximal and distal portions (Figure 1).

### 2.2. Linkage Disequilibrium Analysis

**Figure 2:**
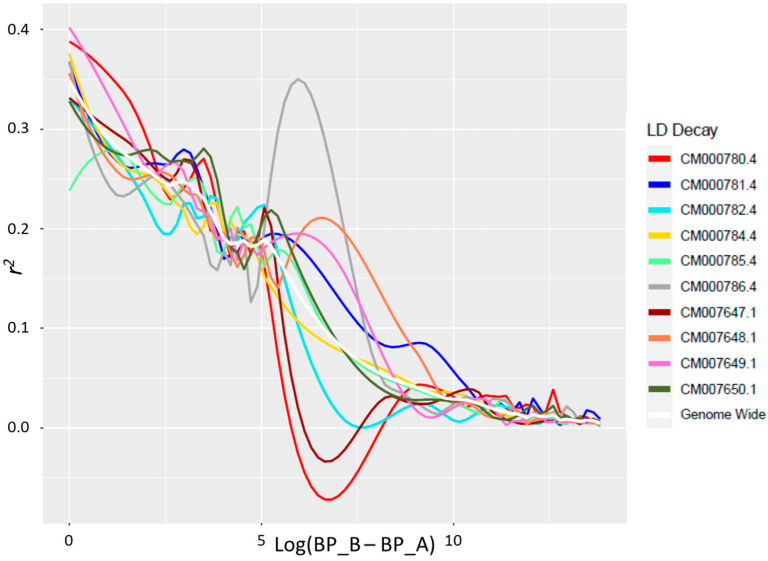
Linkage disequilibrium (LD) decay pattern for all chromosomes and genome-wide level in the entire panel of 360 genotypes. Linkage disequilibrium *r*^2^ is reported on the y-axis, while the log of the distance between the two SNPs for each SNP pair is on the x-axis.

LD decay was evaluated at both the genome-wide and single-chromosome levels. LD patterns changed for the different maize chromosomes and showed some variations for particular chromosomal regions (Figure 2). On average, considering the collection of 360 maize accessions, LD decay occurred at a distance of approximately 12.3 kb, when applying a cut-off value of *r*^2^ = 0.1.

### 2.3. Stratification Analysis of the Maize Collection

The inbreds under study were assigned to groups based on available pedigree data, including ‘Insubria’, ‘Microsperma’, the elite lines derived from US germplasm, and white grain genotypes.

**Figure 3:**
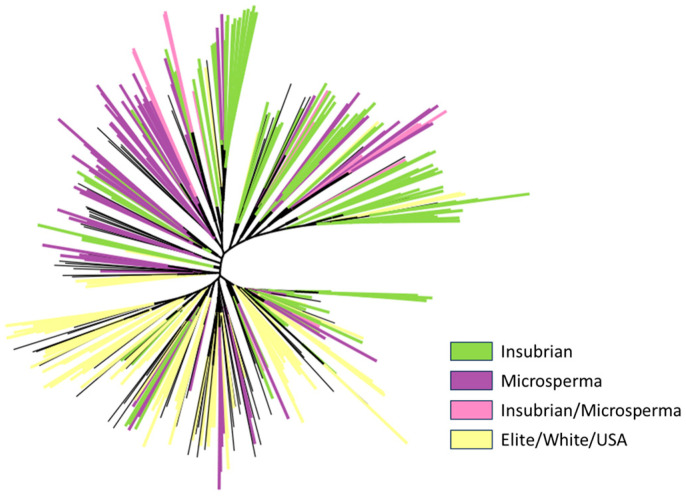
Phylogenetic tree of the maize panel. Black branches represent lines not included in the four main groups.

Subsequently, a phylogenetic tree was constructed. As shown in Figure 3, the majority of the lines belonging to the ‘Insubria’ type, as well as the ‘Microsperma’ lines, were subdivided into two separate groups. Furthermore, a third, more distributed group including the elite lines derived from the US germplasm and white grain genotypes was also present.

**Figure 4:**
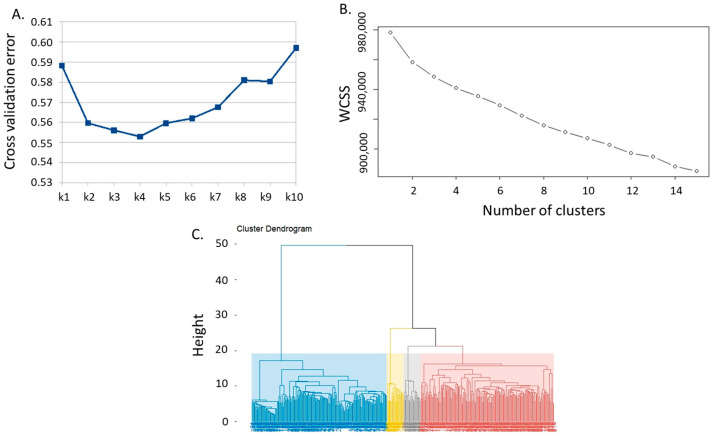
Clustering grouping statistics: (**A**) cross-validation error rate for *ADMIXTURE*; (**B**) within-cluster sum of square for K-means; (**C**) cluster dendrogram showing the division of the four groups (indicated by different colors) for hierarchical clustering.

Population stratification was analyzed using *ADMIXTURE* subpopulation membership from k = 2 up to k = 20 based on the SNP dataset pruned at *r*^2^ = 0.8. Grouping statistics, in particular the cross-validation error rate for *ADMIXTURE*, stabilized at k = 4 and highlighted a certain differentiation between the inbred lines derived from the same cultivar/landrace. The stratification of the collection was also analyzed at K = 4 for the other grouping methods, based on the within-cluster sum of the square for K-means, and the cluster dendrogram showing the division of the four groups was used for hierarchical clustering (Figure 4).

**Figure 5:**
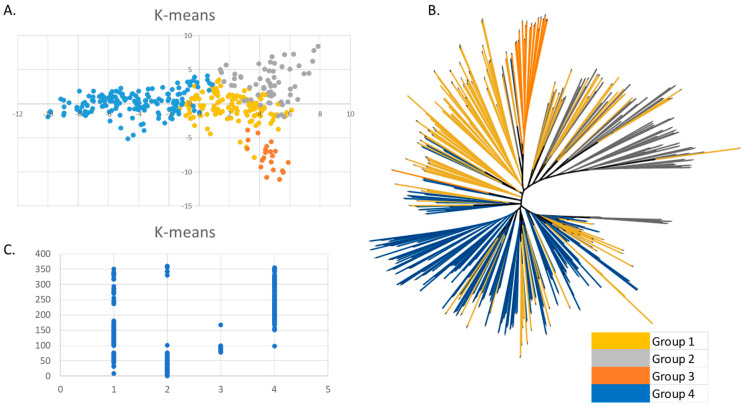
Clustering and phylogenetic analysis of the maize panel: (**A**) population structure generated with K-means combined with PCA; (**B**) phylogenetic tree: colors indicate the groups identified through K-means, in black common sections of branches are reported; (**C**) correspondence between the grouping obtained through K-means and the phylogenetic tree.

A first analysis based on PCA revealed that the two first components collectively explained 5.07% of the total variance (3.5% for the first component and 1.6% for the second). It was possible to identify a certain differentiation between the lines based on these two components, even though the maize lines did not form clearly separate groups (Figure 5).

The results of the three clustering methods, alone or combined with PCA (first five components) or LDA, were compared using a support vector machine algorithm. The results of the comparison showed that the nonparametric clustering algorithms seemed to respond better in the prediction phase than *ADMIXTURE*, with accuracy levels of 0.90 in *ADMIXTURE*, 0.92 in K-means, and 0.92 in hierarchical clustering for raw data; 0.90 in *ADMIXTURE*, 0.94 in K-means, and 0.88 in hierarchical clustering for PCA; and 0.77 in *ADMIXTURE*, 0.84 in K-means, and 0.79 in hierarchical clustering for LDA. Considering the Kappa coefficient, we estimated that the best-performing methods are K-means and hierarchical clustering for raw data (0.86), K-means for PCA (0.9), and K-means for LDA (0.77). According to the comparison made with the support vector machine algorithm, in this specific case, K-means appeared to be the best clustering method, as it differentiated the groups formed by ‘Insubria’, ‘Microsperma’, and the elite/USA/white types better.

Based on the above-mentioned method, Group 1 (119 lines) collection mainly involved the ‘Microsperma’ lines and some ‘Insubria’ lines of the ‘Pignoletto’ type and a few lines derived directly from ‘Nostrano dell’Isola’ or from crosses between one ‘Insubrian’ and one ‘Microsperma’ parent. Group 1 also included 34 lines of miscellaneous type. Group 2 (63 lines) was mostly composed of the ‘Insubria’ lines belonging to the ‘Nostrano dell’Isola’ type and the lines derived from crosses in which one or both parents were ‘Nostrano dell’Isola’. Very few lines of the ‘Cinquantino’ type, the lines derived from crosses with US germplasm, and the lines belonging to the miscellaneous group were present in Group 2. Group 3 (22 lines) contained specific genotypes belonging to the ‘Scagliolino GV’ type, one ‘Nostrano dell’Isola’ and two ‘Marano’. Finally, Group 4 (156 lines) contained the elite lines, white grain genotypes, the lines derived from US germplasm, many ‘Insubria’ lines, a few ‘Microsperma’ lines, and 50 lines from the miscellaneous group (Figure 5A). Interestingly, a certain correspondence between K-means clustering and the phylogenetic tree was observed, as illustrated in Figure 5B,C.

The other two clustering methods yielded similar results, particularly for the very well-separated group of the ‘Scagliolino GV’ type, but some different aspects were also observed (Figure S1).

Based on the *ADMIXTURE* analysis, the first group (68 lines) included a majority of ‘Insubria’ lines, in particular, ‘Nostrano dell’Isola’, ‘Isolabasso’, and ‘Scagliolo’, as well as the lines derived from crosses between ‘Nostrano dell’Isola’ and ‘Isolabasso’ or ‘Marano’. Some ‘Microsperma’ lines, such as ‘Sacra famiglia’ and ‘Cinquantino’, and very few elite and white lines were also included in this group. The second group (87 lines) was mainly composed of ‘Insubria’ lines, in particular, ‘Nostrano dell’Isola’, and some ‘Microsperma’ lines as well as ‘Cinquantino’ and ‘Sacra Famiglia’. Group 3 (68 lines) involved a very well-defined set of ‘Insubria’ lines (‘Scagliolino GV’, the lines derived from the ‘Pignoletto’ group and crosses in which ‘Pignoletto’ was one of the parents), and the ‘Microsperma’ lines belonging to the types ‘Sacra Famiglia’, ‘Marano’, ‘San Pancrazio’, and ‘Cinquantino’. The fourth group (137 lines) comprised in particular the elite lines, the lines derived from US breeding programs, and the lines with white grain. Additionally, some ‘Insubria’ lines, one ‘Nostrano dell’Isola’, some lines derived from crosses between ‘Nostrano dell’Isola’ and other lines in which those derived from USA and ‘Scagliolo’, and a few lines belonging to ‘Scagliolo’ group were included in the fourth group. Finally, a few ‘Microsperma’ lines, in particular, ‘Sacra Famiglia’ and ‘Cinquantino’, were also included in this large group.

According to hierarchical clustering analysis, the first group (44 lines) was composed of the elite lines and some ‘Insubria’ lines belonging to the ‘Nostrano dell’Isola’ type. Group 2 (198 lines) mainly encompassed the ‘Insubria’ and ‘Microsperma’ lines (79 and 62, respectively), besides the other lines of the miscellaneous type. Group 3 (22 lines) specifically contained the ‘Scagliolino GV’ type and just one ‘Nostrano dell’Isola’ and a few ‘Marano’ lines. Group 4 (96 lines) largely corresponded to the *ADMIXTURE* Group 4 (except for ‘Microsperma’ lines’) and contained the elite lines, the lines derived from US breeding programs, white grain genotypes, and the lines derived from crosses between ‘Nostrano dell’Isola’ and US lines or other ‘Insubria’ lines.

### 2.4. Genetic Diversity Analysis

A genetic diversity analysis of the maize collection based on genotyping data was carried out. Differentiation within and among groups based on the group classification obtained through the K-means method was also defined. AMOVA highlighted a low level (3.25%) of genetic variance distinguishing the four groups (Table 2A), with the largest portion being observed within groups (96.75%). In Group 4, containing many modern lines, compared to the groups characterized by inbred lines derived from traditional landraces, a reduction in the overall diversity was observed. Group 3, containing the lines of the ‘Scagliolino GV’ type, was also the most differentiated group in terms of *F_st_* values and was shown to be the one with the highest genetic diversity. Indeed, the *F_st_* values of Group 3 were higher than those of Group 4 and Group 2 (0.078 and 0.054, respectively; Table 2B).

## 3. Discussion

A consistent reduction in crop genetic diversity has been commonly associated with intense breeding activities with the aim of developing better-performing cultivars. Even if breeding has undoubtedly improved the yield and end-product quality of the most important commodities such as maize, likely during the breeding process, some genes/alleles useful for agronomic traits have been lost. For this reason, the maize germplasm collections composed of the inbreds derived from different sources, such as traditional landraces and elite breeding lines, are of paramount importance as they represent the reservoirs of genetic diversity useful to face the current agriculture challenges, mainly linked to the need for increasing production in more severe and variable environmental conditions. In this perspective, the work conducted in the early 1950s by the Regional Inspectorates of Agriculture (*Ispettorati Provinciali dell’Agricoltura*) and the Experimental Station for Maize Cropping (*Stazione Sperimentale per la Maiscoltura*) of Bergamo is of great importance. Samples of maize populations grown in different Italian regions were collected, preserved, and multiplied at Bergamo, to avoid landrace extinction and the loss of precious germplasm. More than 600 inbreds were obtained from these samples, through self-pollination or cross-breeding between the lines derived from landraces and/or lines developed through recent breeding activities. Inbreds are part of a larger maize germplasm collection now conserved at the CREA (Research Centre for Cereal and Industrial Crops of Bergamo). For the present study, a subset of 384 inbreds belonging to different groups was chosen for in-depth genotypic characterization. Table S2 shows some of the available data in terms of phenotypic traits, racial type, and pedigree for those lines derived from a cross-breeding procedure performed at the CREA. As the seed samples were collected nearly seventy years ago, passport data were not available, and therefore it was not possible to reconstruct the exact geographic origin of each seed sample. For these reasons, it was extremely necessary to characterize these materials from a genetic point of view as a first step. Genetic analyses based on molecular markers can indeed provide a precise classification of these lines, with the definition of the genetic relationships across lines to identify homogeneous groups. This information is needed to use this resource in the best way in breeding programs and to develop a core collection with a reduced number of lines expressing the same genetic diversity observed in the whole collection.

Based on the available information, 106 inbreds were assigned to the group ‘Insubria’ (or ‘Padani’), a racial group of inbreds deriving from convergent adaptation to the agrosystem located in the peneplains of the Insubrian–Euganean region of Italy, where maize found a preferential habitat [3]. This class included (i) 41 inbreds derived from ‘Nostrano dell’Isola’, a group of landraces widely grown in many provinces of Italy and originating in the sub-Alpine region of the Bergamo province, and (ii) other lines of different types such as ‘Isola Basso’, ‘Scagliolo’ and ‘Scagliolino’. The inbred types ‘Pignoletto’, ‘Isola Basso’, ‘Rostrato’, ‘Scagliolo’, and ‘Scagliolino’ were found to also belong to the ‘Insubria’ group. A second racial group corresponds to the ‘Microsperma’ type, (‘Microsperma flints’), which includes landraces characterized by subcylindrical ears, small seeds with a very hard and horny texture, medium plant size, and suitable for late spring or summer planting. Seventy inbred lines belong to this group, including those derived from the landraces ‘Marano’, ‘Nostrano dell’Isola maranizzato’, Cinquantino’, ‘Sacra Famiglia’, and ‘San Pancrazio’. Additionally, 15 lines were derived from a cross between an ‘Insubrian’ and a ‘Microsperma’ line. Also, some elite inbreds, developed between 2000 and 2012 as part of the breeding activities carried out at the CREA—Research Centre for Cereal and Industrial Crops of Bergamo (23 lines), were included in the analysis, together with 23 genotypes with white seeds comprising pearl white flints and white dents, and 45 inbreds derived from the lines of the US breeding programs. The remaining lines could not be assigned to a particular group (Table S2).

Choosing the genotyping method is crucial when evaluating large collections of accessions. In this context, an optimal balance between costs, number, and quality of markers has to be considered. SSR markers are still used in genetic diversity studies in maize, as they are codominant and highly informative, having many alleles per locus. Nevertheless, the more recent availability of reference genomes also for species with very complex genomes, as well as the development of next-generation sequencing techniques for generating sequence variation data, has made it possible to develop large sets of SNPs at a relatively low cost for this kind of study. Although SNP arrays show several advantages, such as the presence of exonic and intronic SNPs, markers differentiated between heterotic groups, and markers associated with known genes, they also have limitations. On the other hand, GBS has been improved for SNP discovery and mapping thanks to the two-enzyme approach, and sequence data software and pipelines have recently been developed [23]. GBS has been extensively used for genetic diversity studies, GWAS, and genomic selection approaches in many crops, including maize [24]. It offers the advantage of not having ascertainment bias, although it can be characterized by a high rate of missing data when the sequencing depth is not optimal, thus strongly reducing the number of usable markers when a strict filtering process is applied to retain high-quality markers [23]. The strong reduction in the number of markers was confirmed in the present study, in which nearly 16,000 SNPs were retained from an initial pool of 797,368. Nonetheless, the coverage of the maize genome was good, and an average of 99.72% of the chromosomal extension was tagged by SNPs. Additionally, the physical coverage was in general good, with an average of 7.60 SNPs/Mbp, with some variations between chromosomes (chromosomes 4, 6, and 9 showed a lower coverage) and within chromosomes, with the gene-depleted pericentromeric regions (Figure 1). With a reported gene density in maize of 0.5–10.7 genes per 100 Kbp [25], our dataset could be used for the identification of candidate genes for traits, identified in association studies. All the SNPs used for the different analyses were biallelic, and a certain percentage of heterozygous individuals were identified for each marker. As the accessions of our germplasm collection were inbreds, a filter based on heterozygous loci (20%) was applied to obtain the final SNP dataset, according to the scientific literature [9]. The SNP dataset used in the present study appears suitable for an in-depth genetic characterization of the germplasm maize collection under study in terms of the percentage of missing data, allelic frequency, and heterozygous loci. Moreover, it presents the great advantage of the lack of ascertainment bias usually presented by SNP arrays. Indeed, the SNPs used in the present study were directly developed in the accessions of the germplasm collection analyzed, allowing us to obtain coherent results with different methods of analysis, as described later.

The observed LD decay in the maize collection was 12.3 Kb at *r*^2^ = 0.1. This value is comparable to other studies, as values between a few kb and hundreds of Kb are usually identified at the same value of *r*^2^. As an example, the average decay distance of the LD across all chromosomes was about 5.2 kb at *r*^2^  =  0.1 for a panel of 80 maize inbred lines covering more than 80 % planting area in Jilin Province (China) [26]. Similarly, an LD decay rate of 2.65 Kb at *r*^2^  =  0.1 was found in the CAAM panel (419 tropical/subtropical lines) assembled using CIMMYT to map resistance to northern corn leaf blight [27]. On the other hand, higher values, such as 41.5 kb in a panel of 226 inbreds from China [11] and 310 kb in another Chinese panel of 292 inbred lines [9], were also observed. The LD decay value is very important to assess the resolution power in genome-wide association mapping studies for a germplasm collection. The value identified for our panel indicates that it is suitable for future applications in that sense.

With a total expected heterozygosity of 0.12 and a mean PIC value of 0.225, the 360 entries of the CREA maize collection show a good level of genetic diversity considering their restricted geographical origin. These values are in line with those identified in previous studies carried out with SNP markers. In fact, it is known that SSR markers are characterized by higher PIC values due to their multiallelic nature. Aci et al. [28] found a value of 0.622 in an Algerian maize collection (47 landraces) from Saharan Oasis genotyped with 18 SSRs. Lu et al. [29] analyzed a set of 287 tropical and 160 temperate maize inbred lines, genotyped with 1943 high-quality SNPs, and found a PIC value of 0.251 for the entire set with small differences when the tropical and temperate sets were considered separately. Wu et al. [11] found a significantly higher PIC value (0.60), probably due to the integration of the temperate set with many lines from the Suwan region that are characterized by a larger genetic diversity. Chittò et al. [30] found PIC values between 0.28 and 0.36 for a series of AFLP markers in a set of 71 Italian inbred lines. Similarly, Losa et al. [31] found a PIC value of 0.34 in a collection of 144 Italian inbred lines considered representative of the breeding material developed at the Bergamo Maize Breeding Station and genotyped with 811 AFLP loci.

Grouping statistics stabilized at k = 4, as reported in Figure 4; so, at this k value, it is possible to identify the main landrace types corresponding to the groups ‘Insubria’, ‘Microsperma’, and a large group including more recent elite lines, in which those derived from the US germplasm and white grain genotypes are present, in addition to a smaller group well separated from other lines corresponding to the ‘Scagliolino’ type.

The different methods used in this study provided comparable results in grouping the lines, with some differences, in particular for ‘Microsperma’ lines. These lines were well separated from the other groups using the K-means method. Accordingly, the comparison between the three methods performed with the SVM algorithm also suggested a better performance for K-means. K-means and hierarchical clustering are nonparametric machine learning methods [32,33,34] that are not based on the assumptions of the Hardy–Weinberg principle and use external dimension reduction techniques, such as principal component analysis [35], commonly used in several data-intensive biological fields [36]. Similarly to our results, other authors found that nonparametric methods are more effective than ADMIXTURE in assigning individuals to groups [36].

In general, we did not observe a clear and complete separation of genotypes based on the landrace group name or other phenotypic characteristics reported in Table S2 even if in general lines derived from US breeding programs and from foreign countries were grouped together. As explained above, as the sampling of most lines was carried out many decades ago, it was not possible to reconstruct the exact geographic origin of each seed sample, and this made it difficult to find a precise correspondence between the genetic grouping of lines and their geographical origin. Moreover, the absence of correlation between the genetic variability and the geographic origin of sample provenance could be explained by the fact that most lines were collected in regions of northern Italy, the wider area devoted to maize cultivation, where farmers from different provinces could easily exchange seeds. An example is the ‘Nostrano dell’Isola’ type; it traces back its origin to the Caribbean cylindrical maize types, and some lines are characterized by a medium-late growing cycle and a typical long ear with an enlarged butt and isodiametric orange flint grains [3]. It represents a very large and diverse group in Italy, and similar types are endemic in other maize countries of southern Europe: Portugal, Spain, and Romania. In our study, the lines belonging to the ‘Nostrano dell’Isola’ racial type encompassed at least three groups identified using K-means and the other clustering methods; additionally, they showed a certain degree of differentiation based on their origin. In the maize collection described in the present study, the lines derived from ‘Nostrano dell’Isola’ accessions; the lines derived from crosses in which both parents were ‘Nostrano dell’Isola’; and the lines derived from crosses in which one of the parents was ‘Nostrano dell’Isola’, and the other parent was a line belonging to ‘Microsperma’ or elite lines or lines from USA breeding programs were included. More in detail, most of the lines were derived from crosses in which one of the parents was ‘Nostrano dell’Isola’ and the other parent was a line belonging to ‘Microsperma’, an elite line, or a line from USA breeding programs, clustered in Group 4 identified with K-means, together with elite lines or lines from USA breeding programs. In the same group, a unique ‘Nostrano dell’Isola’ line, which was probably the parent of the other lines, was also included. Inbreds of the ‘Microsperma’ type were mainly clustered in Group 1 identified using K-means, but in this case, there were also some exceptions, such as the accessions Lo440 (‘San pancrazio 85’), Lo495 (‘Marano Vicentino’), and Lo425 (‘Cinquantino San fermo’), which clustered in Group 4, despite being derived from samples with the same name as other ‘Microsperma’ inbreds of Group 1.

The lack of correspondence between clustering and the name of the different Italian lines was also observed in previous studies in which a small subset of this collection was characterized with AFLP markers [30,31]. Another example is provided by some lines belonging to the ‘Pignoletto d’oro’ type: They are positioned with lines of the ‘Microsperma’ group despite ‘Pignolo’ lines being considered of the ‘Insubria’ type. Some ‘Marano’ lines belonging to the ‘Microsperma’ type are very similar to ‘Nostrano dell’Isola’. This finding can be explained by the fact that ‘Marano’ lines are considered to be derived from crosses between ‘Nostrano locale’ lines derived from ‘Nostrano dell’Isola’, grown in 1916 in the Bergamo Province, and ‘Pignoletto d’oro’ [37].

The AMOVA analysis revealed a low level (3.25%) of genetic variance between groups (Table 2), compared to the largest portion observed within groups (96.75%). Similar results have been shown for other maize collections, even if with different proportions of variance, depending on the diversity and heterogeneity of the lines included in the different panels. As an example, in recent studies, values of genetic variance between groups varying from 3% [38] to 17% [39] were reported. Based on Nei’s genetic diversity, a reduction was observed in Group 4, containing many modern lines, compared to groups characterized by inbred lines derived from traditional landraces. This result is in line with the reduction in diversity observed in general following the events of domestication and breeding observed not only in maize but also in other crops such as wheat [40].

The ‘Scagliolino’ type represents a well-defined group for which a clear correspondence is found between clustering and the name of the lines. Moreover, white grain lines also tend to be grouped together, as previously observed in [3]. Genetic diversity analysis is also useful to identify duplicate inbreds in a germplasm collection. The different analyses carried out in the present study allowed us to identify some very similar inbreds, as in the case of Lo3 and Lo16, both derived from ‘Nostrano dell’isola’, and Lo579 and Lo580, both derived from ‘Nostrano dell’isola maranizzato’ samples. This information will be undoubtedly useful in developing a core collection from the current germplasm panel.

A set of 49 Lo Italian lines belonging to our collection and provided by the CREA to INRAE, 28 of which were in common with our present study, were previously included in a large panel of maize accessions derived from different countries by Gouesnard et al. [7]. The Italian lines were derived from different landraces of the ‘Insubria’ group such as ‘Nostrano dell’Isola’, ‘Isolabasso’, and ‘Scagliolo’, and they grouped together in PCoA analysis. Accordingly, the lines of that study with similar results to the results of our present study also grouped together, except in some cases. Of course, the Italian origin of most of the lines in our collection restricts its genetic basis compared to a wider collection composed of lines with a worldwide provenience such as that described by Gouesnard and colleagues [7]. Nonetheless, the genetic variation in the Bergamo collection remains significant. For this reason, the collection of inbred lines described in the present study represents a valuable source of genetic diversity to contribute to maize European breeding programs. Moreover, the available genotypic characterization allows us to exploit the collection in genome-wide association studies. These could be aimed to identify, other than new QTLs, new alleles at the loci of interest for specific traits eventually left behind during the modern breeding process but which can still be useful in breeding, such as genes for resistance to diseases.

## 4. Materials and Methods

### 4.1. Plant Material

The panel involves 384 maize inbreds (coded as ‘Lo’ followed by a numeric value), including 353 lines from a wider panel of lines preserved at CREA Bergamo GenBank and 31 additional advanced breeding lines (elite inbreds—EILs) derived from crosses between different Lo lines (Table S2). These inbreds were mainly derived from samples of landraces cultivated in different regions of northern Italy before the diffusion of hybrids. All the lines belonged to ‘Indentata’ and ‘Indurata’ (dent and vitreous, flint) groups.

### 4.2. Genotyping and Data Processing

The lines were grown in 2018 in Bergamo in the open field. Each genotype was sown in 4 m long rows (20–25 plants/row). Single plants were self-pollinated, and a sample from the flag leaf of each line was collected and air-dried in a vacuum dryer. All the samples were then shipped to Freedom Markers (Ames, IA, USA) for DNA extraction and subsequent analysis. The lines were subjected to extensive SNP search through the tunable genotyping-by-sequencing (tGBS^®^) technology [22] conducted with the restriction enzyme Bsp1286I (Freedom Markers, Data2Bio, IA, USA). Samples were sequenced using an Illumina HiSeq X instrument, and reads were aligned to the *Zea mays* AGPv4 (GCA_000005005.6) reference genome downloaded from NCBI: https://www.ncbi.nlm.nih.gov/assembly/GCF_000005005.2/ (accessed on 22 September 2020). SNP calling was conducted using only those reads that aligned to a single location in the reference genome.

Based on SNP data, 24 genotypes were removed from subsequent analyses due to a high missing rate. The dataset was then filtered based on 20% missing data, 20% heterozygous calls, and a minimum allele frequency > 0.05 [9]. Using the software PLINK 1.9 [41], redundant markers were taken into account by pruning the dataset through the application of a linkage disequilibrium threshold *r*^2^ = 0.99 genome-wide. Redundant markers were then merged into one unique SNP call. Moreover, two additional pruned datasets were generated by applying two different thresholds at *r*^2^ 0.8 and 0.5 to run the population structure analysis. The dataset was subjected to missing data imputation using the Beagle 5 software and applying default parameters [42].

### 4.3. LD Decay

Pairwise marker correlations (*r*^2^ values) were determined using Plink v.1.9 considering the SNP data for each chromosome. LD decay curves were fitted using the nonlinear model described in Rexroad and Vallejo [43]. The fitted regression curves were used to determine critical parameters of marker distances at *r*^2^ = 0.3 and 0.5. The *r*^2^ of unlinked markers (background noise) was estimated as the 95th quantile of *r*^2^ values of markers on different chromosomes (unlinked set). LD was calculated for each marker using the mean *r*^2^ with the 50 nearest markers and then smoothed as one value using the step-sliding window in order to evaluate the local LD value along chromosomes.

### 4.4. Population Structure and Cluster Analysis

A Bayesian method, *ADMIXTURE* [44], and two nonparametric methods, K-means (KM) [45] and hierarchical clustering (HC) [46], were used for clustering analysis. The dataset results pruned at *r*^2^ = 0.99 are shown. For *ADMIXTURE*, the block relaxation algorithm and the quasi-Newton convergence acceleration method were used [44]. For both methods, belonging to a subpopulation was defined for k values increasing from 2 to 10. The optimal number of clusters was estimated based on *ADMIXTURE*’s cross-validated error rate and minimum group size. The clustering methods were used alone or in combination with external dimension reduction techniques, such as principal component analysis (PCA) and linear discriminant analysis (LDA), calculated with Tassel 5.2 [47] and R package MASS, keeping the first 5 components; PC’s 1 and 2 were visualized in a 2D scatter plot with each PC explaining 3.4 and 1.6 percent of the variance, respectively.

A machine learning approach based on the support vector machine model (R package e1071) with a linear kernel was used to compare the results of the clustering analysis, assessing how dimensionality reduction influenced the prediction accuracy and what combination best fitted the data. While the clustering approaches used were unsupervised (no information on clusters was given a priori), the SVM algorithm is a supervised model for classification and regression analysis. A label was associated with each element of the dataset, i.e., a cluster membership class; the entire dataset was then divided into training and test sets (with a ratio of 0.8). The training set was then trained using the SVM, producing a classifier, which was applied to the test set and provided predictions on the clustering classes. Comparing these results with the expected ones, the confusion matrix with the accuracy values of the predictions and Cohen’s Kappa coefficients [48] for all data combinations (raw, PCA, and LDA) and clusters (ADM, KM, and HC) was generated. To better generalize the predictive results and balance the random effect, it was decided to use the k-fold cross-validation technique [49] with k = 5 (R package caret), i.e., the dataset was divided into 5 parts, and a part was alternately chosen as a test set, with the rest used in the training set; the results are represented as the average of the 5-fold predictions.

### 4.5. Phylogenetic Tree

IQ-TREE v2.1.2 [50] was used for phylogenetic analyses, and the PHYLIP format was used for input files. ModelFinder was used to define the model that minimizes the Bayesian information criterion score, and then an ascertainment bias correction model was applied with an ultrafast bootstrap for 10,000 replicates. The command used was iqtree -s input.phy m MFP+ASC -bb 10,000 -seed 1701, where MFP is a model finder, ASC is an ascertainment bias correction, and bb is an ultrafast bootstrap approximation. The online tool Iroki was used to visualize the tree [51].

### 4.6. Analysis of Genetic Diversity within the Maize Collection

AMOVA was carried out to calculate the genetic diversity among and within populations, together with the fixation index (*F_st_*, [52]) and the polymorphism information content (PIC, [53]). The total number of polymorphic loci (N), Nei’s gene diversity [54], and mean number of pairwise differences within populations were calculated, and statistical significance was assessed based on the least significant difference (LSD) at *p <* 0.05. Population differentiation was evaluated based on Nei’s genetic distance [55] and population pairwise *F_st_*. All calculations were performed using the Arlequin 3.5 software [56], and significance levels for variance components and *F_st_* statistics were estimated based on 10,000 and 1000 permutations, respectively.

## Figures and Tables

**Table 1:** Maize chromosomal distribution of filtered SNP markers retained for this study.

Chromosome	SNPs	SNPs/Mb	Chromosome Coverage (%)
1	2315	7.57	99.61
2	1864	7.66	99.57
3	1719	7.32	99.70
4	1631	6.62	99.68
5	1742	7.79	99.85
6	1189	6.85	99.67
7	1391	7.64	99.85
8	1529	8.46	99.78
9	1233	7.73	99.90
10	1259	8.37	99.60
**Total**	**15,872**	**-**	**-**
**Mean**	**1587.2**	**7.60**	**99.72**

**Table 2:** (**A**) AMOVA and (**B**) gene diversity for five germplasm subsets defined according to K-means classification. (**C**) Above-diagonal elements (shades of green) of the matrix contain the average number of pairwise differences, while below-diagonal elements (shades of blue) correspond to pairwise *F_st_* values. Diagonal elements (shades of orange) report the gene diversity within groups calculated as the mean number of pairwise differences. Significance was assessed upon 1000 permutations. All values are significant at *p <* 0.001.

A.				
**Source of variation**	**d.f.**	**Sum of squares**	**Variance components**	**Percentage of variation**
Among populations	3	18,361.74	32.18	3.25
Within populations	716	685,463.41	957.35	96.75
Total	719	703,825.14	989.53	
*F_st_*		0.032		
B.				
**Maize groups**	**N^o^ accessions**	**N^o^ polymorphic loci over 15,872**	**Nei’s gene diversity**	**Mean number of pairwise differences**
K4-1	119	15,868	0.1312	2081.88
K4-2	63	15,243	0.1340	2042.19
K4-3	22	12,864	0.1685	2167.74
K4-4	156	15,857	0.1073	1701.07
Mean value			0.1352	1998.22
LSD (*p* = 0.05)			0.0010	14.97
LSD (*p* = 0.001)			0.0016	23.58
C.				
Group	1	2	3	4
1	2081.882	2090.928	2212.779	1936.086
2	0.014	2042.191	2222.115	1955.579
3	0.041	0.054	2167.744	2078.851
4	0.023	0.045	0.078	1701.074

## Data Availability

The authors confirm that the data supporting the findings of this study are available within the article and its supplementary materials.

## Supplementary Materials

The following supporting information can be downloaded at: https://www.mdpi.com/article/10.3390/plants13030336/s1, Figure S1: Clustering and phylogenetic analysis of the maize panel. (A) Population structure generated with ADMIXTURE combined with PCA. (B) Phylogenetic tree: the colors indicate the groups identified through ADMIXTURE. (C) Correspondence between the grouping obtained using ADMIXTURE and the phylogenetic tree. (D) Population structure generated with K-means combined with PCA. (E) Phylogenetic tree: the colors indicate the groups identified using K-means. (F) Correspondence between the grouping obtained with K-means and the phylogenetic tree. (G) Population structure generated with hierarchical clustering combined with PCA. (H) Phylogenetic tree: the colors indicate the groups identified through hierarchical clustering. (I) Correspondence between the grouping obtained through hierarchical clustering and the phylogenetic tree; Table S1: Number of quality-trimmed sequence reads and aligned reads for each sample. Table S2: Origin, morpho-phenological characteristics, population stratification, and PCA analysis of the maize panel.

## References

[1] (2023). ISTAT. https://dati.istat.it/Index.aspx?DataSetCode=DCSP_COLTIVAZIONI.

[2] Ardenghi N.M.G., Rossi G., Guzzon F. (2018). Back to beaked: *Zea mays* subsp. mays Rostrata Group in northern Italy, refugia and revival of open-pollinated maize landraces in an intensive cropping system. Peer J..

[3] Brandolini A., Brandolini A. (2009). Maize introduction, evolution and diffusion in Italy. Maydica.

[4] Levi G. (2014). The Diffusion of Maize in Italy: From Resistance to the Peasants’ Defeat. Global Goods and the Spanish Empire, 1492–1824: Circulation, Resistance and Diversity.

[5] Brandolini A. (1958). Il germoplasma del mais e la sua conservazione. Maydica.

[6] Evers T., Millar S. (2002). Cereal Grain Structure and Development: Some Implications for Quality. J. Cereal Sci..

[7] Gouesnard B., Negro S., Laffray A., Glaubitz J., Melchinger A., Revilla P., Moreno-Gonzalez J., Madur D., Combes V., Tollon-Cordet C. (2017). Genotyping-by-sequencing highlights original diversity patterns within a European collection of 1191 maize flint lines, as compared to the maize USDA genebank. Theor. Appl. Genet..

[8] Ganal M.W., Durstewitz G., Polley A., Bérard A., Buckler E.S., Charcosset A., Clarke J.D., Graner E.-M., Hansen M., Joets J. (2011). A large maize (*Zea mays* L.) SNP genotyping array: Development and germplasm genotyping, and genetic mapping to compare with the B73 reference genome. PLoS ONE.

[9] Zhu X.-M., Shao X.-Y., Pei Y.-H., Guo X.-M., Li J., Song X.-Y., Zhao M.-A. (2018). Genetic diversity and genome-wide association study of major ear quantitative traits using high-density SNPs in maize. Front. Plant Sci..

[10] Negro S.S., Millet E.J., Madur D., Bauland C., Combes V., Welcker C., Tardieu F., Charcosset A., Nicolas S.D. (2019). Genotyping-by-sequencing and SNP-arrays are complementary for detecting quantitative trait loci by tagging different haplotypes in association studies. BMC Plant Biol..

[11] Wu X., Wang A., Guo X., Liu P., Zhu Y., Li X., Chen Z. (2019). Genetic characterization of maize germplasm derived from Suwan population and temperate resources. Hereditas.

[12] Hu H., Schrag T.A., Peis R., Unterseer S., Schipprack W., Chen S., Lai J., Yan J., Prasanna B.M., Nair S.K. (2016). The genetic basis of haploid induction in maize identified with a novel genome-wide association method. Genetics.

[13] Millet E.J., Welcker C., Kruijer W., Negro S., Coupel-Ledru A., Nicolas S.D., Laborde J., Bauland C., Praud S., Ranc N. (2016). Genome-wide analysis of yield in Europe: Allelic effects vary with drought and heat scenarios. Plant Physiol..

[14] Unterseer S., Bauer E., Haberer G., Seidel M., Knaak C., Ouzunova M., Meitinger T., Strom T.M., Fries R., Pausch H. (2014). A powerful tool for genome analysis in maize: Development and evaluation of the high density 600 k SNP genotyping array. BMC Genom..

[15] Messing J., Dooner H.K. (2006). Organization and variability of the maize genome. Curr. Opin. Plant Biol..

[16] Frascaroli E., Schrag T.A., Melchinger A.E. (2013). Genetic diversity analysis of elite European maize (*Zea mays* L.) inbred lines using AFLP, SSR, and SNP markers reveals ascertainment bias for a subset of SNPs. Theor. Appl. Genet..

[17] Glaubitz J.C., Casstevens T.M., Lu F., Harriman J., Elshire R.J., Sun Q., Buckler E.S. (2014). TASSEL-GBS: A high capacity genotyping by sequencing analysis pipeline. PLoS ONE.

[18] Le Gouis J., Bordes J., Ravel C., Heumez E., Faure S., Praud S., Galic N., Remoué C., Balfourier F., Allard V. (2012). Genome-wide association analysis to identify chromosomal regions determining components of earliness in wheat. Theor. Appl. Genet..

[19] Bauer E., Falque M., Walter H., Bauland C., Camisan C., Campo L., Meyer N., Ranc N., Rincent R., Schipprack W. (2013). Intraspecific variation of recombination rate in maize. Genome Biol..

[20] Springer N.M., Ying K., Fu Y., Ji T., Yeh C.-T., Jia Y., Wu W., Richmond T., Kitzman J., Rosenbaum H. (2009). Maize Inbreds exhibit high levels of copy number variation (CNV) and presence/absence variation (PAV) in genome content. PLoS Genet..

[21] Cormier F., Le Gouis J., Dubreuil P., Lafarge S., Praud S. (2014). A genome-wide identification of chromosomal regions determining nitrogen use efficiency components in wheat (*Triticum aestivum* L.). Theor. Appl. Genet..

[22] Ott A., Liu S., Schnable J.C., Yeh C.T.E., Wang K.S., Schnable P.S. (2017). tGBS^®^ genotyping-by-sequencing enables reliable genotyping of heterozygous loci. Nucleic Acids Res..

[23] Elshire R.J., Glaubitz J.C., Sun Q., Poland J.A., Kawamoto K., Buckler E.S., Mitchell S.E. (2011). A robust, simple genotyping-by-sequencing (GBS) approach for high diversity species. PLoS ONE.

[24] Tiwari A., Choudhary S., Padiya J., Ubale A., Mikkilineni V., Char B., Sonah H., Goyal V., Shivaraj S.M., Deshmukh R.K. (2022). Recent advances and applicability of GBS, GWAS, and GS in maize. Genotyping by Sequencing for Crop Improvement.

[25] Haberer G., Young S., Bharti A.K., Gundlach H., Raymond C., Fuks G., Butler E., Wing R.A., Rounsley S., Birren B. (2005). Structure and architecture of the maize genome. Plant Physiol..

[26] Moussa A.A., Mandozai A., Jin Y., Qu J., Zhang Q., Zhao H., Anwari G., Khalifa M.A.S., Lamboro A., Noman M. (2021). Genome-wide association screening and verification of potential genes associated with root architectural traits in maize (*Zea mays* L.) at multiple seedling stages. BMC Genom..

[27] Rashid Z., Sofi M., Harlapur S.I., Kachapur R.M., Dar Z.A., Singh P.K., Zaidi P.H., Vivek B.S., Nair S.K. (2020). Genome-wide association studies in tropical maize germplasm reveal novel and known genomic regions for resistance to Northern corn leaf blight. Sci. Rep..

[28] Aci M.M., Lupini A., Mauceri A., Morsli A., Khelifi L., Sunseri F. (2018). Genetic variation and structure of maize populations from Saoura and Gourara oasis in Algerian Sahara. BMC Genet..

[29] Lu Y., Shah T., Hao Z., Taba S., Zhang S., Gao S., Liu J., Cao M., Wang J., Prakash A.B. (2011). Comparative SNP and haplotype analysis reveals a higher genetic diversity and rapider LD decay in tropical than temperate germplasm in maize. PLoS ONE.

[30] Chittò A., Bertolini M., Hartings H., Verderio A., Motto M. (2000). AFLP-based genetic relationships among maize inbred lines selected in a climatically temperate location. Maydica.

[31] Losa A., Hartings H., Verderio A., Motto M. (2012). Assesment of genetic diversity and relationships among maize inbred lines developed in Italy. Maydica.

[32] Bouaziz M., Paccard C., Guedj M., Ambroise C. (2012). SHIPS: Spectral hierarchical clustering for the inference of population structure in genetic studies. PLoS ONE.

[33] Meirmans P.G. (2012). AMOVA-based clustering of population genetic data. J. Hered..

[34] Alhusain L., Hafez A.M. (2018). Nonparametric approaches for population structure analysis. Hum. Genet..

[35] Kobak D., Berens P. (2019). The art of using t-SNE for single-cell transcriptomics. Nat. Commun..

[36] López-Cortés X.A., Matamala F., Maldonado C., Mora-Poblete F., Scapim C.A. (2020). A Deep Learning Approach to Population Structure Inference in Inbred Lines of Maize. Front. Genet..

[37] Brandolini A., Brandolini A., Negri A., Maggiore T., Angelini R. (2008). Origine e diffusione. Il Mais.

[38] Ayesiga S.B., Rubaihayo P., Oloka B.M., Dramadri I.O., Edema R., Sserumaga J.P. (2023). Genetic variation among tropical maize inbred lines from NARS and CGIAR breeding programs. Plant Mol. Biol. Rep..

[39] Naveenkumar K.L., Sen D., Vashum S., Sanjenbam M. (2020). Genetic characterization and divergence studies of maize (*Zea mays* L.) lines developed from landraces indigenous to North Eastern Hill Region (NEHR) of India. Plant Genet. Res. Charact. Util..

[40] Mazzucotelli E., Sciara G., Mastrangelo A.M., Desiderio F., Xu S.S., Faris J., Hayden M.J., Tricker P.J., Ozkan H., Echenique V. (2020). The Global Durum Wheat Panel (GDP): An international platform to identify and exchange beneficial alleles. Front. Plant Sci..

[41] Chang C.C., Chow C.C., Tellier L.C., Vattikuti S., Purcell S.M., Lee J.J. (2015). Second-generation PLINK: Rising to the challenge of larger and richer datasets. Gigascience.

[42] Browning B.L., Zhou Y., Browning S.R. (2018). A one-penny imputed genome from next-generation reference panels. Am. J. Human Genet..

[43] Rexroad C.E., Vallejo R.L. (2009). Estimates of linkage disequilibrium and effective population size in rainbow trout. BMC Genet..

[44] Alexander D.H., Novembre J., Lange K. (2009). Fast model-based estimation of ancestry in unrelated individuals. Genome Res..

[45] MacQueen J.B. (1967). Some methods for classification and analysis of multivariate observations. Proceedings of the 5-th Berkeley Symposium on Mathematical Statistics and Probability.

[46] Kaufman L., Rousseeuw P.J. (1990). Finding Groups in data: An Introduction to Cluster Analysis.

[47] Bradbury P.J., Zhang Z., Kroon D.E., Casstevens T.M., Ramdoss Y., Buckler E.S. (2007). TASSEL: Software for association mapping of complex traits in diverse samples. Bioinformatics.

[48] Cohen J. (1960). A coefficient of agreement for nominal scales. Educ. Psychol. Meas..

[49] Stone M. (1974). Cross-validatory choice and assessment of statistical predictions. J. R. Stat. Soc. Ser. B.

[50] Minh B.Q., Schmidt H.A., Chernomor O., Schrempf D., Woodhams M.D., Von Haeseler A., Lanfear R. (2020). IQ-TREE 2: New models and efficient methods for phylogenetic inference in the genomic era. Mol. Biol. Evol..

[51] Moore R.M., Harrison A.O., McAllister S.M., Polson S.W., Wommack K.E. (2020). Iroki: Automatic customization and visualization of phylogenetic trees. Peer J..

[52] Wright S. (1965). The interpretation of population structure by F-statistics with special regard to systems of mating. Evolution.

[53] Botstein D., White R.L., Skolnick M., Davis R.W. (1980). Construction of a genetic linkage map in man using restriction fragment length polymorphisms. Am. J. Hum. Genet..

[54] Nei M. (1973). Analysis of gene diversity in subdivided populations. Proc. Natl. Acad. Sci. USA.

[55] Nei M. (1972). Genetic distance between populations. Am. Nat..

[56] Excoffier L., Lischer H.E.L. (2010). Arlequin suite ver 3.5: A new series of programs to perform population genetics analyses under Linux and Windows. Mol. Ecol. Resour..

