# Supplementary material for: Genetic Diversity within a Collection of Italian Maize Inbred Lines: A Resource for Maize Genomics and Breeding
Source: Plants (Basel). 2024 Jan 23;13(3):336. doi: 10.3390/plants13030336 (PMC10857507; doi:10.3390/plants13030336)

Figure S1. Clustering and phylogenetic analysis of the maize panel. A. Population structure carried out with *ADMIXTURE* combined with PCA. B. Phylogenetic tree: the colors indicate the groups identified through *ADMIXTURE*. C. Correspondence between the grouping obtained through *ADMIXTURE* and the phylogenetic tree. D. Population structure carried out with K-means combined with PCA. E. Phylogenetic tree: the colors indicate the groups identified through K-means. F. Correspondence between the grouping obtained through K-means and the phylogenetic tree. G. Population structure carried out with hierarchical clustering combined with PCA. H. Phylogenetic tree: the colours indicate the groups identified through hierarchical clustering. I. Correspondence between the grouping obtained through hierarchical clustering and the phylogenetic tree.

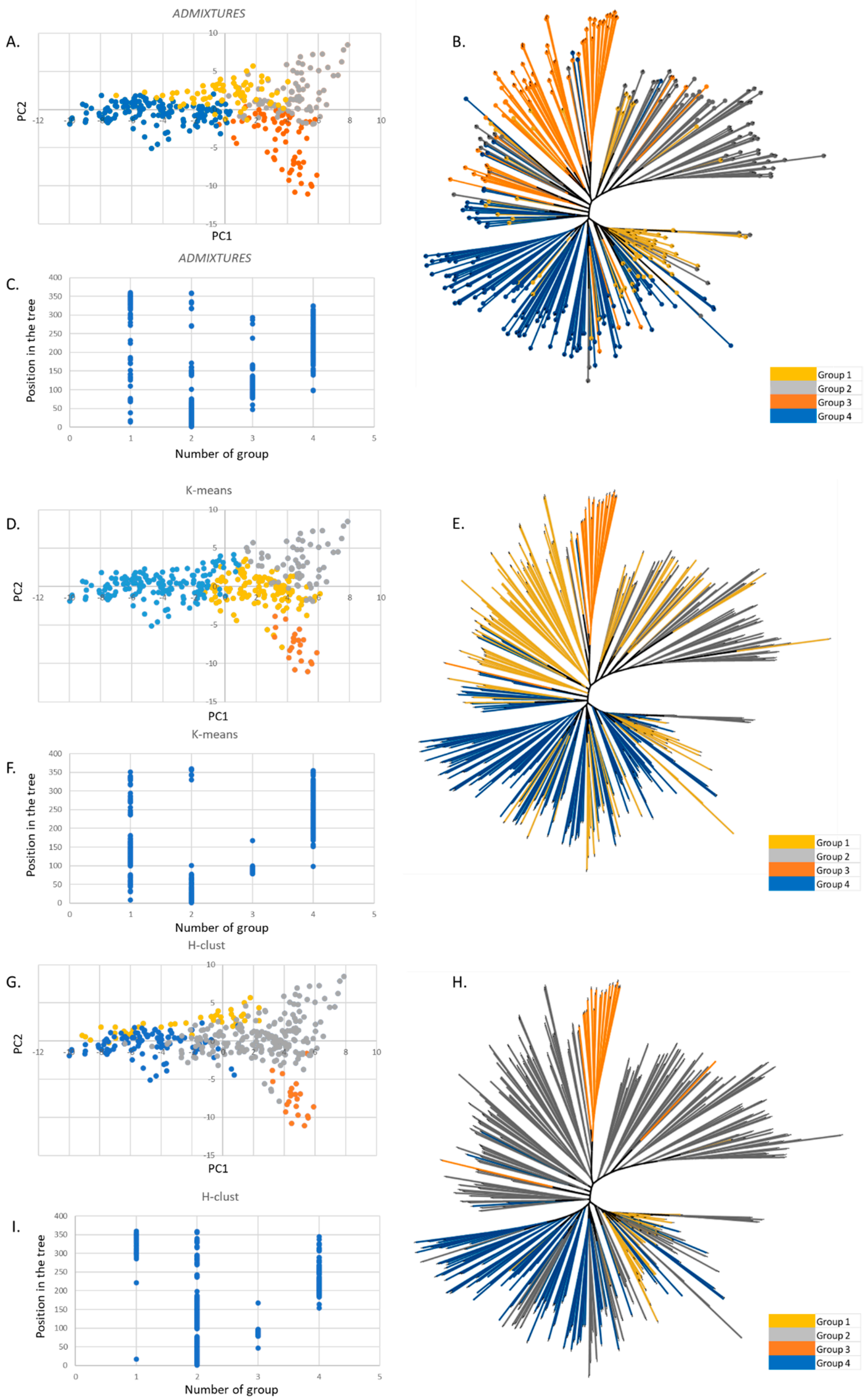

Supplement: Supplementary file 1 [file plants-13-00336-s001.zip › Supplementary Figure S1.pdf]
